# A Simple and Highly Effective Method for Slow-Freezing Human Pluripotent Stem Cells Using Dimethyl Sulfoxide, Hydroxyethyl Starch and Ethylene Glycol

**DOI:** 10.1371/journal.pone.0088696

**Published:** 2014-02-12

**Authors:** Keitaro Imaizumi, Naoki Nishishita, Marie Muramatsu, Takako Yamamoto, Chiemi Takenaka, Shin Kawamata, Kenichiro Kobayashi, Shin-ichi Nishikawa, Teruo Akuta

**Affiliations:** 1 Laboratory for Stem Cell Biology, RIKEN Center for Developmental Biology, Minatojima-Minamimachi, Chuo-ku, Kobe, Hyogo, Japan; 2 Kobe office, RIKEN Cell Tech Co. Ltd., Minatojima-Minamimachi, Chuo-ku, Kobe, Hyogo, Japan; 3 Division of Cell Therapy, Foundation for Biomedical Research and Innovation, Minatojima-Minamimachi, Chuo-ku, Kobe, Hyogo, Japan; 4 Department of Pediatric Hematology and Oncology Research, National Research Institute for Child Health and Development, Okura, Setagaya-ku, Tokyo, Japan; University of Tampere, Finland

## Abstract

Vitrification and slow-freezing methods have been used for the cryopreservation of human pluripotent stem cells (hPSCs). Vitrification requires considerable skill and post-thaw recovery is low. Furthermore, it is not suitable for cryopreservation of large numbers of hPSCs. While slow-freezing methods for hPSCs are easy to perform, they are usually preceded by a complicated cell dissociation process that yields poor post-thaw survival. To develop a robust and easy slow-freezing method for hPSCs, several different cryopreservation cocktails were prepared by modifying a commercially available freezing medium (CP-1™) containing hydroxyethyl starch (HES), and dimethyl sulfoxide (DMSO) in saline. The new freezing media were examined for their cryopreservation efficacy in combination with several different cell detachment methods. hPSCs in cryopreservation medium were slowly cooled in a conventional −80°C freezer and thawed rapidly. hPSC colonies were dissociated with several proteases. Ten percent of the colonies were passaged without cryopreservation and another 10% were cryopreserved, and then the recovery ratio was determined by comparing the number of Alkaline Phosphatase-positive colonies after thawing at day 5 with those passaged without cryopreservation at day 5. We found that cell detachment with Pronase/EDTA followed by cryopreservation using 6% HES, 5% DMSO, and 5% ethylene glycol (EG) in saline (termed CP-5E) achieved post-thaw recoveries over 80%. In summary, we have developed a new cryopreservation medium free of animal products for slow-freezing. This easy and robust cryopreservation method could be used widely for basic research and for clinical application.

## Introduction

Human induced pluripotent stem cells (hiPSCs) [1] and human embryonic stem cells (hESCs) [2] are both classified as human pluripotent stem cells (hPSCs). They have received great attention for their potential pharmaceutical applications and therapeutic use in regenerative medicine (reviewed in [3]). In addition, patient-specific hiPSCs have been generated for a variety of diseases [4]. Analysis of hPSCs will improve our understanding of human diseases and advance the field toward clinical applications. There are, however, several hurdles to overcome. One of them is the need for a robust cryopreservation method. Many studies have been conducted to establish an efficient, economical and robust cryopreservation method using animal-free components [5].

Two distinct cryopreservation methods have been developed for hPSCs, namely, vitrification and slow-freezing methods. Vitrification has been reported elsewhere [6]–[10]. Cryopreservation media with high cryoprotectant concentrations are used for vitrification and hPSCs are rapidly frozen with liquid nitrogen. This technique requires skill and is not suitable for cryopreservation of large amounts of hPSCs [10]. In contrast, cryopreservation by slow-freezing methods does not require special skills [11]. After centrifugation, hPSCs are resuspended in a cryopreservation medium followed by gradual freezing in a deep freezer or programmable freezer. This method has allowed us to freeze large amounts of hPSCs, but low post-thaw recoveries compared with vitrification have been a distinct drawback. As a result, an anti-apoptotic reagent (Rho-associated kinase (ROCK) inhibitor, Y-27632) has often been used in the freezing/thawing process [12].

Several cryoprotective agents have been used to minimize cellular damage during the freezing process. The most common substance is dimethyl sulfoxide (DMSO) [13], [14]. Combinations of various protective reagents such as trehalose [15], ethylene glycol (EG) [16], PEG [17], high polymer (STEM-CELLBANKER™) [18], hydroxyethyl starch (HES), and plant-derived hydrolysate [19], [20] have been used with DMSO in slow freezing protocols.

In this article, we examined 5 different cryopreservation cocktails, modifying a commercially available freezing medium CP-1™ (Kyokuto Pharmaceutical Industrial, Tokyo Japan). Sixty eight mL of CP-1™ consists of 12 g of HES and 10 mL of DMSO in saline. CP-1™ has been used in Japan for the cryopreservation of cord blood stem cells and bone marrow stem cells for over 20 years [21]–[24]. In current practice, CP-1™ is mixed just before use with 25% human serum albumin (HSA) solution. Then, cells from a single cord blood unit (≥1×10^9^ nucleated cells) suspended in RPMI1640 culture medium are added, yielding the final freezing medium [6% HES, 5% DMSO, 4% HSA, and 50% RPMI1640 in saline]. CP-1™ is a safe and low-cost cryopreservation medium that is easy to prepare. Therefore we tested whether addition of other reagents to the final CP-1™ formulation could enhance the recovery rate after thaw.

In studies of hPSC preservation, it is also important to optimize the method of cell detachment because the size of cell clumps and retention of surface molecules after enzyme digestion are crucial for post-thaw survival. Five different cell detachment reagents (collagenase IV [19], [25], [26], Dispase II [6], 0.05% trypsin/EDTA [27], [28], CTK solution [29], and Pronase/EDTA [30], [31]) were tested in combination with modified CP-1™ freezing medium.

Finally, we used a conventional −80°C freezer for overnight cooling (omitting a programmable freezer [32]) to determine whether our protocol could be simplified for general use.

## Materials and Methods

### Cell culture

hiPSC lines [201B7 [1] and 253G1 [33] (Riken BRC, Tsukuba, Japan)], and hESC lines [KhES-1 [34] (Riken BRC) and H1 [2] (WiCell)] were used in these experiments. These cell lines were cultured on mitomycin C-treated SNL76/7 [European Collection of Cell Culture (ECACC), cat.no. 07032801, lot no. 08F009] in 6-well tissue culture plates (BD Biosciences) at 37°C in an atmosphere of 5% CO_2_ with hPSC culture medium [Dulbecco's modified Eagle's medium (D-MEM)/F12 (Life Technologies) supplemented with 20% knockout serum replacement (KSR: Life Technologies), 2 mM GlutaMax™ (Life Technologies), 1% non-essential amino acids (Life Technologies), 0.1 mM 2-mercaptoethanol (Wako, Osaka, Japan), 5 ng/mL basic fibroblast growth factor (Wako), and penicillin-streptomycin (Meiji, Tokyo, Japan)]. Passage of cells was initiated by washing with phosphate-buffered saline [PBS (-)] followed by incubation at 37°C in CTK dissociation solution [PBS (-) containing 0.25% trypsin, 1 mg/mL collagenase IV (Invitrogen), 20% KSR, and 1 mM CaCl_2_ (Nacalai Tesque, Kyoto, Japan)] [35]. When SNLs detached and the edge of hiPSC/ESC colonies started curling, CTK solution was removed and cells were washed once with the culture medium. Cells were then collected by gentle pipetting, and transferred to a new dish pre-coated with SNL76/7. The culture medium was changed every day, and the split ratio was routinely 1∶6 every 4 days to give a similar number of cell colonies after every passage.

### Cell detachment

Five different cell dissociation solutions were evaluated to determine which yielded the best recovery of hPSCs. The solutions included the following: 1) Pronase/EDTA [0.075 mg/mL Pronase, 0.6 mM EDTA in PBS (-) (Kyokuto Pharmaceutical Industrial, Tokyo, Japan)], 2) 0.05% trypsin/EDTA (Invitrogen), 3) 1× Dispase II (Roche; 10× solution was diluted with D-MEM/F12 medium), 4) 1.5 mg/mL collagenase IV (Invitrogen; powder was reconstituted with D-MEM/F12 medium), or 5) CTK. Under all conditions, hPSCs cultured in 6-well culture plates (approx. 300 colonies, total 250,000 cells in a well) were dissociated, and SNL feeder cells were removed by aspiration. Colonies after dissociation were collected in 15 mL tubes by adding 5 mL of hPSC culture medium. Ten percent of total cells (25,000 cells) were passaged and anoher 10% were cryopreserved to determine the recovery ratio after thawing. Even though the numbers of cells cultured per well were almost equal, the number of colonies varied depending on the enzymatic treatment because each treatment resulted in generating clumps of different sizes. The incubation times for enzymatic treatment were as follows: 2 min for Pronase/EDTA, trypsin/EDTA, and CTK. In contrast, 1× Dispase II required 20 min and 1.5 mg/mL collagenase IV required 40 min respectively. All treatments were conducted in an incubator at 37°C in 5% CO_2_.

### Cell cryopreservation

After brief centrifugation (300×g for 3 min), cell clumps consisting of some 25,000 cells were resuspended in 0.5 mL of ice-cold freezing cocktail (A, B, C, D, or E, defined below). Cocktails were prepared from CP-1™ basal freezing medium [total volume 68 mL of saline containing 12 g of hydroxyethyl starch (HES) and 10 mL of DMSO, Kyokuto Pharmaceutical Industrial]. Final concentrations of cocktail constituents of A–E were as follows:

**A**, [6% (w/v) HES, 5% (v/v) DMSO, 4% (w/v) bovine serum albumin (BSA; Sigma), and 50% (v/v) D-MEM/F12 in saline]

**B**, [6% (w/v) HES, 5% (v/v) DMSO, and 50% (v/v) D-MEM/F12 in saline]

**C**, [6% (w/v) HES, 5% (v/v) DMSO, and 4% (w/v) BSA in saline]

**D**, [6% (w/v) HES and 5% (v/v) DMSO in saline]

**E**, [6% (w/v) HES, 5% (v/v) DMSO, and 5% (v/v) ethylene glycol (EG; WAKO) in saline]

Cells were then transferred to 2-mL cryovials (AGC, Tokyo, Japan) and immediately placed into a freezing container (NALGENE™ Cryo 1°C Freezing Container; Nalgene, USA) for cooling in a −80°C freezer. After storage in a −80°C freezer overnight, the vials were stored for at least 1 week in a −150°C freezer (Sanyo) prior to thawing for the evaluation of recovery rate. All freezing media were prepared just before use.

### Thawing and recovery

Cryovials were warmed in a water bath at 37°C for approximately 30 sec until the icy mass disappeared, and then the cell suspension was diluted by addition of 5 mL of 37°C hPSCs culture medium. After the supernatant was removed by centrifugation (300×g, 3 min), cell pellets were resuspended in 2 mL of fresh hPSC culture medium and cultured in a 6-well plate with pre-seeded SNL feeders [35]. For the first 2 days of culture, cells were cultured in medium supplemented with 10 µM Y-27632 [12]. From the third day of culture onward, hPSC culture medium was changed daily without Y-27632. The post-thaw recovery frequencies (rate, %) were calculated as follows. hPSC colonies were dissociated with the indicated proteases by gently pipetting 5 times and 10% of the colonies were passaged without cryopreservation and another 10% were cryopreserved. On day 5 of incubation, cultured colonies (without cryopreservation) or post-thaw colonies were subjected to alkaline phosphatase (ALP) staining. The percentage post-thaw ALP+ colonies was compared with those that had simply been maintained in culture. All experiments were repeated in triplicate. The Tukey test was performed to evaluate statistical differences. A *P* value <0.05 was considered significant.

### hPSC clump size analysis

Digital images of hPSC clumps were acquired after cell detachment to measure horizontal areas of the clumps with the BioRevo microscope system (Keyence). One hundred hPSC clumps were randomly selected towards this end. The data were analyzed by a Kruskal-Wallis test followed by a Mann-Whitney U test with Bonferroni correction as post-hoc test.

### hPSC: expansion profile

The number of hiPSC (201B7) (25,000 cells/vial) were scored for 3 passages after thawing or without cryopreservation. Cells were cultured in a 100 mm dish (BD Biosciences) for 7 days just after thawing. Then, colonies were detached with CTK and transferred to a new 6-well plate in a 1∶6 split ratio. Cell numbers in the wells were counted with a hemacytometer after another digesting with 0.05% trypsin/EDTA dissociation buffer. The total cell count was calculated at every passage.

### *In vitro* differentiation

hPSCs (201B7 and KhES-1) clumps harvested with CTK solution were transferred to ultra-low attachment 6-well plates (Corning) to demonstrate their 3 germ layer differentiation potential by forming embryoid bodies (EBs). Cell clumps were incubated in the culture medium without bFGF and the medium was changed every other day. After 8 days of cultivation, EBs were transferred to 0.1% gelatin-coated 6-well plates for RNA extraction or 24-well plates for immunostaining, and continuously cultured in the same medium for another 8 days to differentiate.

Expression of lineage-specific genes was examined by quantitative RT-PCR. Molecules representing 3 germ layer differentiation were detected by immunostaining using antibodies against β-tubulin (ectoderm), β-smooth muscle actin (α-SMA; mesoderm), or α-fetoprotein (AFP; endoderm).

### ALP staining and immunostaining

The activity of ALP was visualized after fixation with 4% (w/v) paraformaldehyde (PFA) in PBS (-) using alkaline phosphatase substrate kit IV (Vector laboratories, CA) per the manufacturer's instruction. Immunocytochemistry was performed in accordance with the Cell Signaling Technology's manual; colonies of hiPSC (201B7) and hESC (KhES-1) were fixed with 4% PFA in PBS (-) for 15 min at room temperature, followed by washing with PBS (-) (5 min×3). When staining for Oct4, ice-cold methanol was added to the cells followed by incubation at −20°C for 10 min for permeabilization. The fixed cells were preincubated with 0.3% Triton X-100 and 5% goat serum (Sigma) in PBS (-) for 1 h at room temperature and then washed once with PBS (-). Expression of Oct4, stage-specific embryonic antigen-3 (SSEA-3), SSEA-4, TRA-1-60, or TRA-1-81 in cultured cells was detected with anti-human-Oct4 (Santa Cruz Biotech, diluted 1∶200), -SSEA-3 (Millipore, diluted 1∶200), -SSEA-4 (Cell Signal Technologies, diluted 1∶200), -TRA-1-60 (CST, diluted 1∶200), or -TRA-1-81(CST, diluted 1∶200) antibody, respectively and visualized with a second antibody labeled with Alexa Fluor 488 (Invitrogen, diluted 1∶500). For the detection of AFP, β-tubulin and α-SMA molecules in differentiated tissue, anti-AFP (CST, diluted 1∶100), anti-β-tubulin (Sigma, diluted 1∶200) or anti-α-SMA (Sigma, diluted 1∶100) antibody were used and then visualized with secondary antibodies labeled with Alexa Fluor 488 or Alexa Fluor 546 (Invitrogen, diluted 1∶500). Nuclei were counterstained with DAPI (Invitrogen). BioRevo fluorescent microscope imaging system (Keyence, Osaka, Japan) was used for fluorescent observation.

### Quantitative RT-PCR

Total RNA was extracted using an RNeasy micro kit (QIAGEN), according to the manufacturer's instructions. Five hundred ng of total RNA were used to synthesize cDNA with QuantiTect Reverse Transcription Kit (QIAGEN). Then, quantitative PCR (qPCR) was performed with SYBR green PCR Master Mix (Life Technologies) and analyzed with a StepOnePlus™ Real-Time PCR System (Life Technologies). The sequences of all primers for pluripotent and lineage-specific genes are listed in **Table S2**. Gene expression was normalized to that of *GAPDH* as the internal control and quantified by the ΔΔCt method.

### Flow cytometric analysis

hiPSC (201B7) and hESC (KhES-1) cells were harvested with CTK and dissociated to single cells with Accutase™ (STEMCELL Technologies). The cells were washed once in PBS (-) containing 2% HSA (Mitsubishi-Tanabe Pharmaceuticals, Japan). A total of 5×10^5^ cells were incubated with the same buffer containing 1/50 volume of the designated fluorescently labeled antibody for 30 min at 4°C. The cells were analyzed with a FACS Aria II (BD Biosciences) after washing once in PBS (-). AlexaFluor647-conjugated anti-SSEA-3, AlexaFluor647-conjugated anti-SSEA-4, and BrilliantViolet421-conjugated anti-TRA-1-60 (all antibodies from BD Biosciences) were used in flow cytometric analysis.

### Teratoma assay

Animal studies were reviewed and approved by FBRI Animal Experiment Committee prior to the study. For teratoma formation assay, 1 million hiPSC (201B7) that had been cultured for 5 passage after thawing were transplanted under the epidermal space of the left testis of NOG mice (CLEA Japan, Tokyo, Japan). Ten µL of saline was injected into the right testis as a negative control (n = 3). Ten weeks after transplantation, all the mice developed teratomas at the injection site. Teratomas formed within the testicles were fixed with 4% formalin (Wako), and sliced sections were stained with hematoxylin (Sakura Finetek, Japan) and eosin (Merck Millipore).

### Karyotyping and CGH array

Karyotyping service was provided by Nihon Gene Research Laboratories, Inc., (Sendai, Japan). Briefly, hiPSC (201B7) were treated with colcemid (Sigma) and were harvested by treatment with 0.25% trypsin/EDTA. Cells were fixed on slides with Carnoy's solution and soaked in Giemsa stain solution (Merck). After washing with water, 50 metaphase spreads were screened and 20 of them were evaluated for chromosomal rearrangements by microscopy (Eclipse E600; NIKON) at 1000× magnification for G-band analysis. For multi-color fluorescein *in situ* hybridization (M-FISH), fixed 201B7 cells were hybridized with a 24XCyte M-FISH probe kit (MetaSystems) and observed by fluorescent microscopy (DM6000B; LEICA) at 1000× magnification.

Array Comparative Genomic Hybridization (CGH) was performed with a SureTag Complete DNA Labeling Kit and SurePrint G3 Human CGH microarray 1×1 M (Agilent Technologies). Briefly, 500 ng of KhES-1 DNA before/after cryopreservation were digested with restriction enzymes *Alu*I and *Rsa*I and labeled with a fluorescence tag, Cy3- or Cy5-dUTP, respectively. The labeled samples along with human Cot-1 DNA were added together and hybridized on the array slides. The slides were scanned at 3 µm resolution on SureScan microarray scanner (Agilent Technologies). Data were quantified with Agilent CytoGenomics software and analyzed with the Genomic Workbench Software (Agilent Technologies).

## Results

### Detaching cells with Pronase/EDTA gave the best post-thaw recovery of hPSCs

The development of a cryopreservation medium for hPSC must be accompanied by an optimal cell detachment method. The method determines the size of cell clumps and the retention of surface adhesion molecules following cell release. Five different cell detachment methods were examined in combination with various cryopreservation media, and post-thaw recoveries were compared (**Figure S1**). We optimized the hPSC dissociation method using hiPSC line 201B7 and freezing Formula A [6% HES, 5% DMSO, and 4% BSA, and 50% D-MEM/F12 in saline]. The rationale for using freezing Formula A was as follows. Starting with this freezing medium, we changed RPMI1640 to D-MEM/F12 because we meant to cryopreserve adherent hPSCs, not suspension cells like cord blood cells. Also, HSA was changed to BSA because hiPSC had been adapted to culture medium containing BSA in KSR.

**Figure 1:**
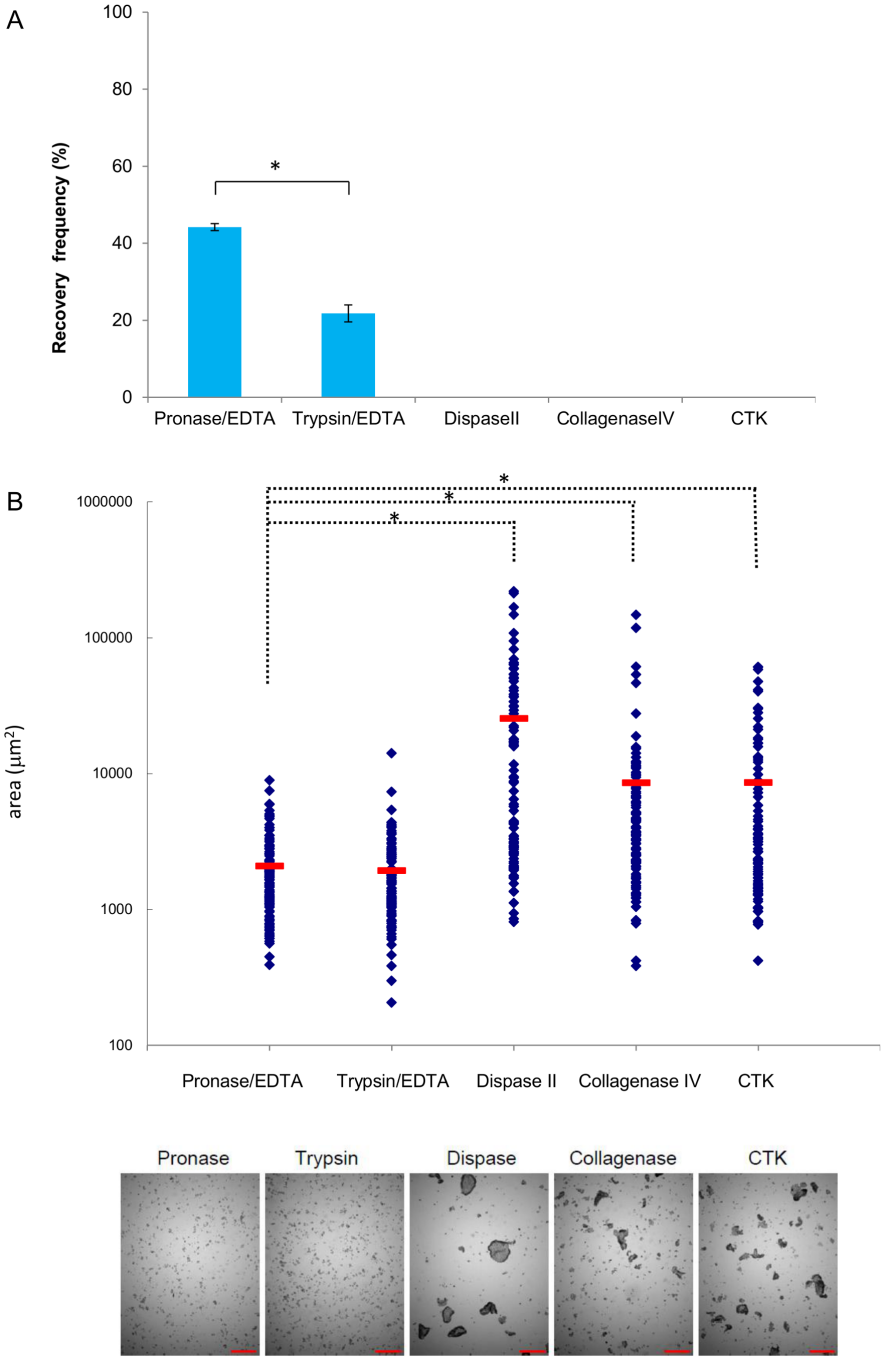
Selection of a cell dissociation reagent suitable for slow-freezing. (A) hiPSC 201B7 colonies were dissociated with Pronase/EDTA, trypsin/EDTA, Dispase II, collagenase IV, or CTK, followed by cryopreservation with Formula A medium (6% HES, 5% DMSO, 4% BSA, and 50% D-MEM/F12 in saline). Recovery frequencies (rate, %) were determined by scoring the number of post-thaw ALP+ colonies at day 5 in 6-well dish for comparison against non-frozen cells at day 5 in 6-well dishes that had been passaged with the same dissociation buffer. The results of 3 independent experiments are shown with standard deviation bars [SD]). *; *P*<0.05. (B) Sizes of 100 randomly selected cell clumps (in µm^2^) after dissociation in the indicated medium. Sizes are plotted as blue dots. Red bars show each median. A photo of cell clumps after use of the indicated cell detachment reagent is shown in the lower panel. Scale bars; 500 µm. *; *P*<0.05.

Five different cell dissociating reagents (Pronase/EDTA, trypsin/EDTA, Dispase II, Collagenase IV, and CTK) were used in combination with cryopreservation medium A. Recoveries were calculated by scoring ALP-positive colony numbers at day 5 post-thaw and comparing with that at day 5 with colony passage with respective dissociation buffer (without cryopreservation). The recoveries with different dissociation reagents are shown in **Figure 1A** and **Table S1**. Pronase/EDTA cell dissociation yielded the highest recovery (44%), whereas trypsin/EDTA yielded only 21% and others resulted in no recovery (*P*<0.05). The sizes of the cell clumps after cell detachment were measured, because surface area of cell clump contacting cryopreservation medium varies by the cell clump size and consequently efficacy of the freezing medium may vary by the size of the cell clumps. We noted that Pronase/EDTA and trypsin/EDTA treatment produced small sized cell clumps (some 2000 µm^2^) (**Figure 1B**). Relatively uniform clump size was obtained by Pronase/EDTA treatment (**Figure 1B** **lower panel**).

### Cryopreservation with CP-1™-based medium supplemented with ethylene glycol yielded good post-thaw recovery

**Figure 2:**
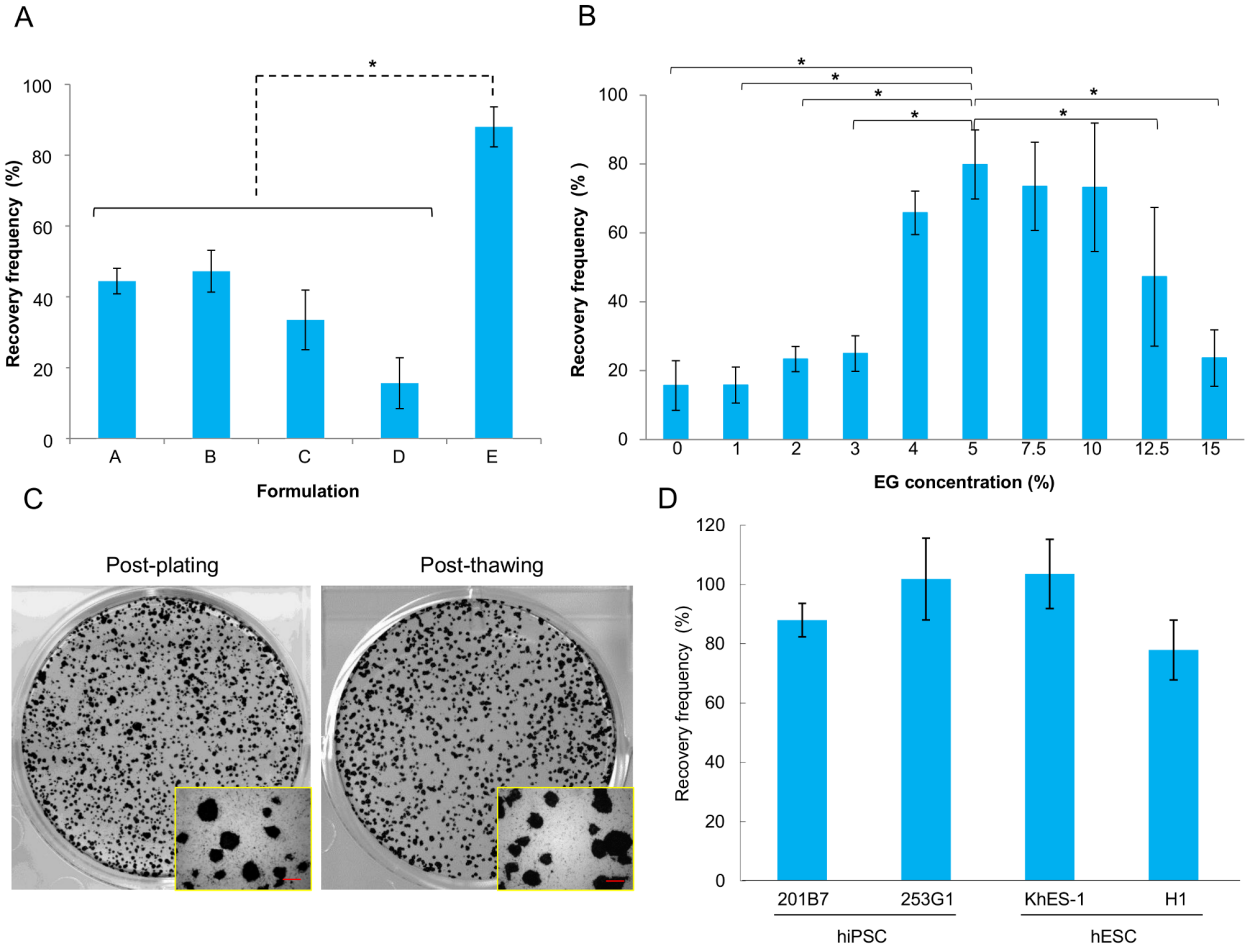
Selection of cryopreservation medium for slow-freezing. (A) Recovery frequencies (rate, %) of iPSC (201B7) colonies treated with Pronase/EDTA dissociation followed by cryopreservation with 5 different media (Formulas A–E). Recovery frequencies (rate, %) were determined by the percentage of ALP+ colonies 5 days after thawing compared with those at day 5 after passaging with Pronase/EDTA without cryopreservation. Recovery frequencies (rate, %) are shown as bars with S.D. Formula A: [6% HES, 5% DMSO, 4% BSA, and 50% D-MEM/F12 in saline]; B: [6% HES, 5% DMSO, and 50% D-MEM/F12 in saline]; C: [6% HES, 5% DMSO, and 4% BSA in saline]; D: [6% HES and 5% DMSO in saline]; E: [6% HES, 5% DMSO, and 5% ethylene glycol (EG) in saline]. Results of 3 independent experiments are shown. Differences between E and the others are significant. *; *P*<0.05. (B) The effects of EG addition on cryopreservation efficacy of freezing media. Various concentrations (1, 2, 3, 4, 5, 7.5, 10, 12.5, or 15% v/v) of EG were added to cryopreservation Formula D (6% HES, 5% DMSO in saline). Recovery frequencies (rate, %) were determined by scoring the post-thaw number of ALP+ colonies and those without cryopreservation. Results of 3 independent experiments are shown. *; *P*<0.05 (C) ALP staining of colonies of iPSC 201B7 maintained for 5 days after passage (left photo: post-plating, non-frozen control) and those 5 days after thaw (right photo: post-thawing, dissociation with Pronase/EDTA and cryopreservation with CP-5E). Magnified photos are attached. Scale bars indicate 500 µm. (D) Cell colonies of hiPSC cell lines (201B7, 253G1) or hESC cell lines (KhES-1, H1) were dissociated with Pronase/EDTA, followed by cryopreservation with CP-5E (Formula E: 6% HES, 5% DMSO, and 5% EG in saline). Recovery frequencies (%) were determined by scoring the number of ALP+ colonies after thawing for comparison with nonfrozen cells. Results of 3 independent experiments are shown.

**Figure 3:**
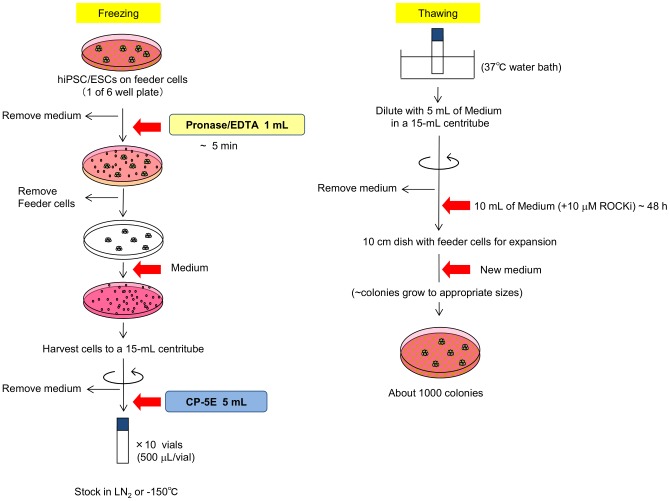
Schematic overview of the protocol for hPSCs cryopreservation and thaw. Schema shows the protocol for the slow-freezing procedure with the combined use of Pronase/EDTA and cryopreservation medium CP-5E (left) and rapid thawing (right).

Next, the cryopreservation potentials of several CP-1™-based media were examined in combination with Pronase/EDTA dissociation. Five cryopreservation media were prepared as described in Materials and Methods, and their efficacies were determined by assessing the frequency of post-thaw ALP-positive colonies (see Materials and Methods) (**Figure 2A**). Formula E yielded the highest recovery rate, suggesting that addition of EG greatly improved the recovery of cryopreserved hPSCs when compared to the results obtained with Formula D, 6% HES and 5% DMSO in saline (*P*<0.05). Also, neither BSA nor D-MEM/F12 appeared to be critical for a high recovery. This result led us to define the optimal concentration of EG in cryopreservation medium by adding various doses (0–15%) of EG to Formula D (**Figure 2B**). A cryopreservation medium containing 6% HES, 5% DMSO and 5% EG in saline, termed CP-5E, showed the greatest efficacy based on the post-thaw recovery of ALP-positive colonies (**Figure 2C**). Post-thaw recoveries of the human iPSC line 253G1 or human ESC lines H1 or KhES-1 after cell dissociation with Pronase/EDTA and cryopreservation by CP-5E were similar, with 80 to 100% recoveries (**Figure 2D**). These data suggested that many hiPSC and hESC cell lines could be cryopreserved and thawed satisfactory using this protocol. The overall scheme of the finally optimized protocol is shown in **Figure 3**.

### Post-thaw characterization of hPSCs

**Figure 4:**
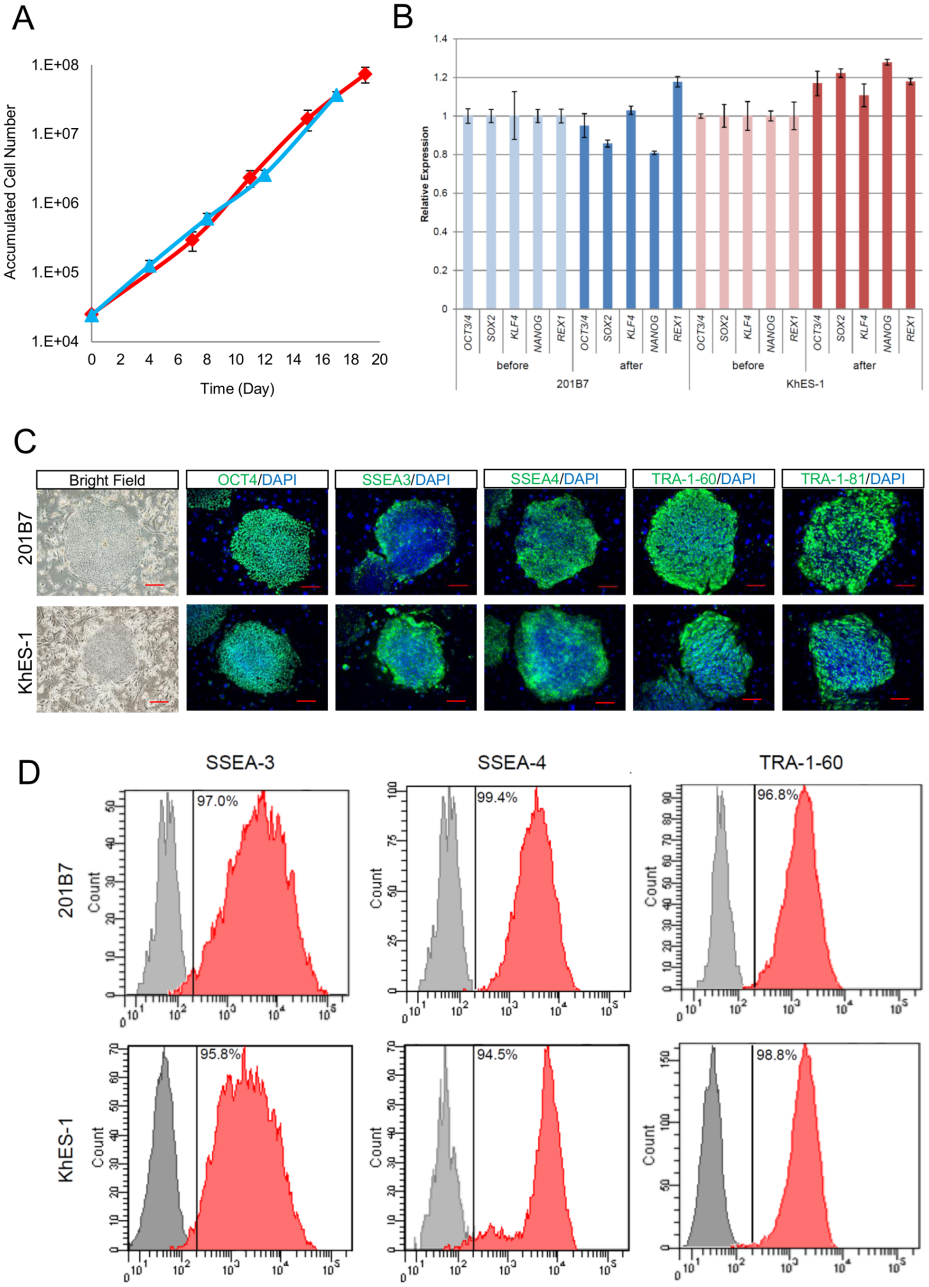
hPSCs retained self-renewal potential and pluripotency after cryopreservation with CP-5E. (A) Cell growth of hiPSC (201B7) before (blue line) and after (red line) thaw. Up to 3 passages (18–20 days) are shown. The experiments were performed in triplicate. (B)hiPSCs (201B7) or hESCs (KhES-1) were cryopreserved with CP-5E. Expression of pluripotency-related transcription factor genes (*OCT4, KLF4, SOX2, NANOG, and REX1*) before and 3 passages after thaw were determined by qRT-PCR. (C)Immunostaining of pluripotency-related molecules (OCT4, SSEA-3, SSEA-4, TRA-1-60, and TRA-1-81) in hiPSCs (201B7) or hESCs (KhES-1) after thawing. These molecules were detected by specific antibodies and visualized with secondary Alexa Fluor 488 (green)-labeled antibody. Nuclei were stained with DAPI. Scale bars, 200 µm. (D) Flow cytometric analysis of pluripotency-related surface markers (SSEA-3, SSEA-4, and TRA-1-60) in hiPSC (201B7) or hESC (KhES-1) after thawing.

Cell growth curves of hiPSC (201B7) before and after cryopreservation with Pronase/EDTA and CP-5E were comparable during 3 passages (20 days) of cultivation (**Figure 4A**). Cryopreservation with CP-5E did not reduce the cells' growth potential after thawing. The pluripotencies of recovered hiPSC (201B7) and hESC (KhES-1) were determined by qRT-PCR, immunocytochemical analysis and flow cytometric analysis. The gene expression profiles of pluripotency–related genes (*OCT4, KLF4, SOX2, NANOG*, and *REX1*) in hiPSC and hESC 3 passages after thawing were not altered (**Figure 4B**). hiPSC and hESC colonies after thawing stained positively for Oct4, SSEA-3, SSEA-4, TRA-1-60, and TRA-1-81 (**Figure 4C**). Flow cytometric analysis showed that the majority of hiPSC (201B7) and hESC (KhES-1) expressed pluripotency-related surface markers SSEA3, SSEA4 and TRA-1-60 (**Figure 4D** **)**. These results suggested that cryopreservation with CP-5E did not alter the post-thaw pluripotencies of hiPSCs and hESCs.

**Figure 5:**
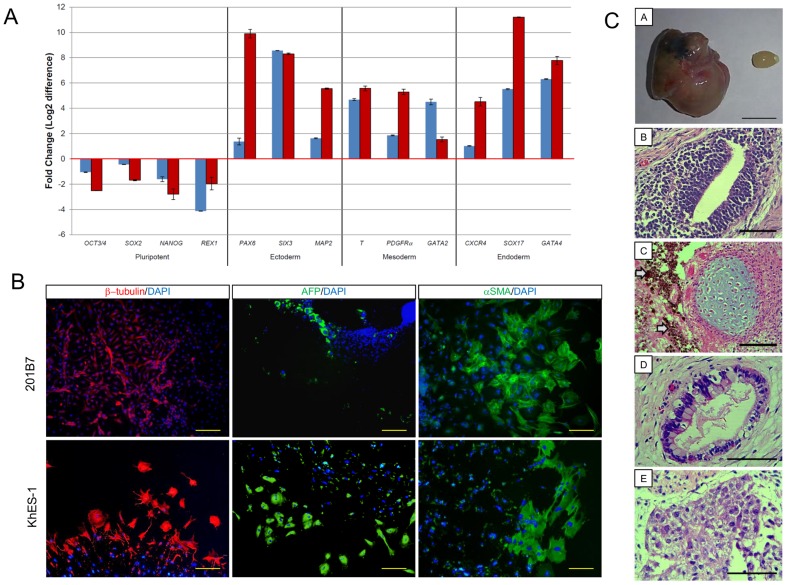
hPSCs maintained differentiation potential after cryopreservation with CP-5E. (A) hPSCs were cryopreserved with CP-5E. Differentiation of hiPSC (201B7) (blue bar) and hESC (KhES-1) (red bar) was initiated via EB formation after thawing. qRT-PCR was used to assess pluripotency–related genes (*OCT4*, *SOX2*, *NANOG*, and *REX1*) and 3 germ layer differentiation marker genes (ectodermal [*PAX6*, *SIX3*, and *MAP2*], mesodermal [*T*, *PDGFRα*, and *GATA2*] and endodermal [*CXCR4*, *SOX17*, and *GATA4*]) before and after thawing. Gene expression before and after differentiation were compared by theΔΔCt method. (B) Differentiation of hiPSC (201B7) and hESC (KhES-1) 5 passages after thawing was initiated via EB formation. Molecules related to 3 germ layer differentiation: β-tubulin (ectoderm), α-SMA (mesoderm), or AFP (endoderm) were detected with specific antibodies and visualized with secondary antibodies labeled with Alexa Fluor 488 (green) or Alexa Fluor 546 (red). Nuclei were stained with DAPI. Scale bars: 200 µm. (C) Assessment of post-thaw teratoma formation by hiPSCs. One million hiPSC (201B7) cells cultured for 5 passages after thawing were transplanted under the epidermal space of the left testes of NOG mice; saline was injected in the right testes of the mice as controls. Ten weeks after transplantation, all mice developed teratomas (n = 3). A: photo of a teratoma (left) and control testis (right). Scale Bar: 1 cm. (B–E) Histological analysis of teratoma. Sections were stained with hematoxylin and eosin. B: neural rosette (ectoderm), C: cartilage (mesoderm) and pigmented melanocytes (arrow heads), D: gut-like epithelium (endoderm), E: immature hepatocyte-like cells (endoderm). Scale Bars: 100 µm.

The *in vitro* multilineage differentiation potentials of hiPSC (201B7) and hESC (KhES-1) 5 passages after thawing were evaluated by qRT-PCR. Genes related to endodermal, mesodermal, or ectodermal differentiation were induced whereas expression of pluripotency-related genes was reduced markedly (**Figure 5A**), suggesting that 201B7 retained 3 germ layer differentiation potential after thawing. This differentiation potential was confirmed by immunostaining with 3 germ layer markers (**Figure 5B**). Furthermore, after thawing, 201B7 retained the ability to form teratomas in NOG mice. Histological analysis of the teratomas showed the distinctive 3 germ layer structure (**Figure 5C**). These results suggested that cryopreservation with CP-5E did not alter the differentiation potentials of hiPSCs and hESCs after thaw.

**Figure 6:**
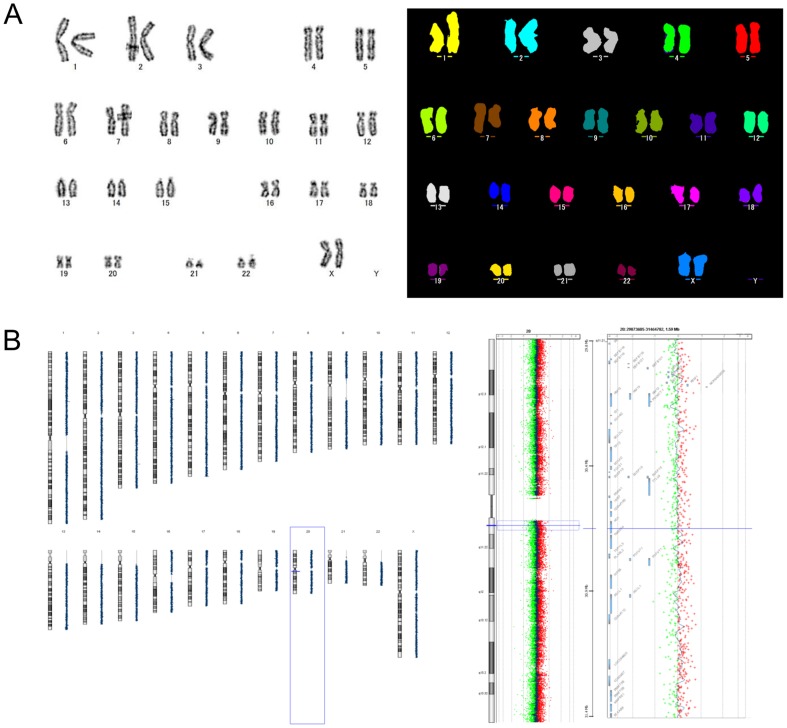
Karyotypic analysis of hPSCs after cryopreservation with CP-5E. (A) Post-thaw G-band analysis (left) and multi-color FISH characterization (right) of hiPSC (201B7, XX). Normal karyotype of 201B7 was observed. 201B7 was cultured for 27 passages before cryopreservation with CP-5E and karyotype analysis was conducted 5 passages after thawing. (B) CGH array analysis of hESC (KhES-1) before and after cryopreservation. Genomic DNA samples of KhES-1 before or 5 passages after cryopreservation were labeled with a fluorescent tag, Cy3 (green dots) or Cy5 (red dots) respectively, and hybridized on an array slide. Fluorescent intensities of Cy3 shown as minus values and Cy5 as plus, and their mean at designated loci in all chromosomes are shown as a black dot in the left panel. There is no distinct difference in fluorescent signal intensity between the 2 samples, suggesting no obvious alteration of chromosomal structure after cryopreservation. Scanning data of whole Chr 20 and the vicinity of 20q11.21 known as a hot spot for CNV are shown in middle and right panels, respectively. KhES-1 was cultured for 33 passages in the lab before cryopreservation and cultured for another 5 passages after thawing.

Finally, karyotypic analyses of 201B7 by G-band and multicolor FISH were conducted pre- and post-thaw. Furthermore, CGH array analyses of KhES-1 before and after cryopreservation were conducted. We could not find any chromosomal structural abnormality in 201B7 or KhES-1 cryopreserved with CP-5E by these methods (**Figure 6**).

## Discussion

Here, we report a new animal component-free and protein-free cryopreservation medium optimized for slow-freezing of hPSCs. It is termed CP-5E (final concentrations: 6% HES, 5% DMSO, and 5% EG in saline). Maximal efficacy was achieved when combined with Pronase/EDTA for cell detachment. Recovery frequencies of hPSCs after thawing were above 80%. This novel method has the following advantages. 1) Cell detachment with Pronase/EDTA requires less than 5 min. 2) Both Pronase/EDTA and CP-5E are relatively inexpensive. 3) Freezing of cells with this process is simple and does not require intensive training. 4) There is no need for a programmable freezer. 5) Rapid thawing in a water bath is simple and does not require any special post-thaw recovery solutions.

Recently, T'joen, et al. [19] reported a similar slow-freezing medium containing 5% HES, 5% DMSO, and freezing vehicle [80% D-MEM/F12+20% knockout serum replacement (KSR) +HEPES] for the cryopreservation of small hESC clumps. Although this protocol is able to handle large numbers of hESCs, it has several shortcomings. Specifically, it contains an animal component from KSR, requires a complex 2-step 30 min dissociation procedure, necessitates use of a programmable freezer and requires the use of sucrose in the post-thaw recovery solution. In this context, our novel cryopreservation method for hPSCs using Pronase/EDTA for dissociation and CP-5E for cryopreservation is much simpler.

Development of the new cryopreservation medium is coupled with an optimized cell dissociation method, as the dissociation process determines the size of cell clumps. Pronase/EDTA dissociated the hPSC colonies into small cell clumps. Pronase is isolated from the extracellular medium of *Streptomyces griseus* cultures [30], [31]. Pronase dissociates hPSC by detaching the SNL feeder cells from the hPSCs and EDTA breaks hPSCs colonies into small clumps. We assume that the small cell clump size obtained with the combination of Pronase/EDTA (some 2000 µm^2^) facilitates good delivery of cryopreservatives to individual cells within the cell clumps. This is the first time that Pronase has been evaluated as a cell dissociation solution in hPSCs culture systems and freezing systems. Dissociation with trypsin/EDTA gave similar sized clumps, but the overall recovery of hPSCs after dissociation with trypsin/EDTA was almost half of that with Pronase/EDTA. It is possible that potent trypsin/ETDA treatment digested key surface molecules or extracellular matrix components that are related to hPSCs cell death during freezing and thawing processes and adhesion after plating.

CP-5E was developed from CP-1™ cryopreservation medium. This simple formula minimizes the risks of exposure to xenogeneic pathogens and lot-to-lot variations arising from differing qualities of BSA. HES is a high molecular weight cryoprotectant derived from a plant and cannot pass through the cell membrane. HES, therefore, remains in the extracellular space and is thought consequently to stabilize the cell membrane [23], [24]. HES has been used as a plasma volume expander and drug stabilizer, suggesting the biological safety of HES. In contrast, DMSO is a low molecular weight compound (78.13 Da) that penetrates the cellular membrane and prevents the formation of ice crystals during cooling or warming. DMSO has been widely used for freezing media, but it is reported to cause hypotonic damage at around 4°C [14]. EG is also a small molecule (62.07 Da) and is widely used as a cryoprotectant. The mode of action of EG is not fully understood, but we assume that addition of EG could attenuate osmotic shock caused by DMSO at around 4°C and consequently contribute to good recovery by preventing apoptotic cell death after thaw. Indeed, even though the recovery of hPSCs cryopreserved with formula D without EG (6% HES and 5% DMSO in saline) was below 20%, that of CP-5E (6% HES, 5% DMSO, and 5% EG in saline) was increased to 80%.

Freezing and thawing processes are stressful events for cells. For that reason, use of ROCK inhibitor (ROCKi), Y-27632, is appealing as it protects cells from apoptosis. ROCKi could be added before cryopreservation and just after the thawing process. Mollamohammadi *et al.* reported that exposure to Y-27632 before dissociation enhanced the survival rate of hPSCs in a feeder-free culture system [27]. However, in our on feeder culture system, 1 hour incubation with Y-27632 prior to dissociation did not increase the post-thaw survival rate of iPSC (201B7). In fact, it decreased the post-thaw number of hiPSC colonies by 20%. The addition of ROCKi during the thawing process increases the survival of hPSCs in both feeder-dependent and feeder indepenedent conditons [36]-[39]. For that reason, we added Y-27632 in the thawing process.

In conclusion, our slow-freezing cryopreservation method allows us to store hPSCs effectively, easily, safely, and economically, and it can be widely used for basic research and the banking of clinical grade hiPSCs/hESCs in the future.

## Supporting Information

Figure S1 **Overview of the protocol for cell passage and the selection of the optimal dissociation buffer for slow-freezing.** Schema shows the protocol for regular hPSCs passage with CTK (1∶6 split ratio, left) and selection of the optimal dissociation buffer for slow-freezing (1∶10 split with various dissociation buffers, right). The 1∶10 split ratio was used for this comparison study to score the number of colonies from small cell clumps after trypsin/EDTA or Pronase/EDTA dissociation.
(TIF)

Table S1 **hiPSC 201B7 colony numbers after thawing using various cell detachment methods.** Scored colony numbers for Figure 1 is shown. Number in the table is the mean ± SD of 3 independent experiments.
(TIF)

Table S2 **List of primers used for quantitative RT-PCR.**
(TIF)
