# Supplementary figures and images for: A Simple and Highly Effective Method for Slow-Freezing Human Pluripotent Stem Cells Using Dimethyl Sulfoxide, Hydroxyethyl Starch and Ethylene Glycol
Source: PLoS One. 2014 Feb 12;9(2):e88696. doi: 10.1371/journal.pone.0088696 (PMC3922972; doi:10.1371/journal.pone.0088696)

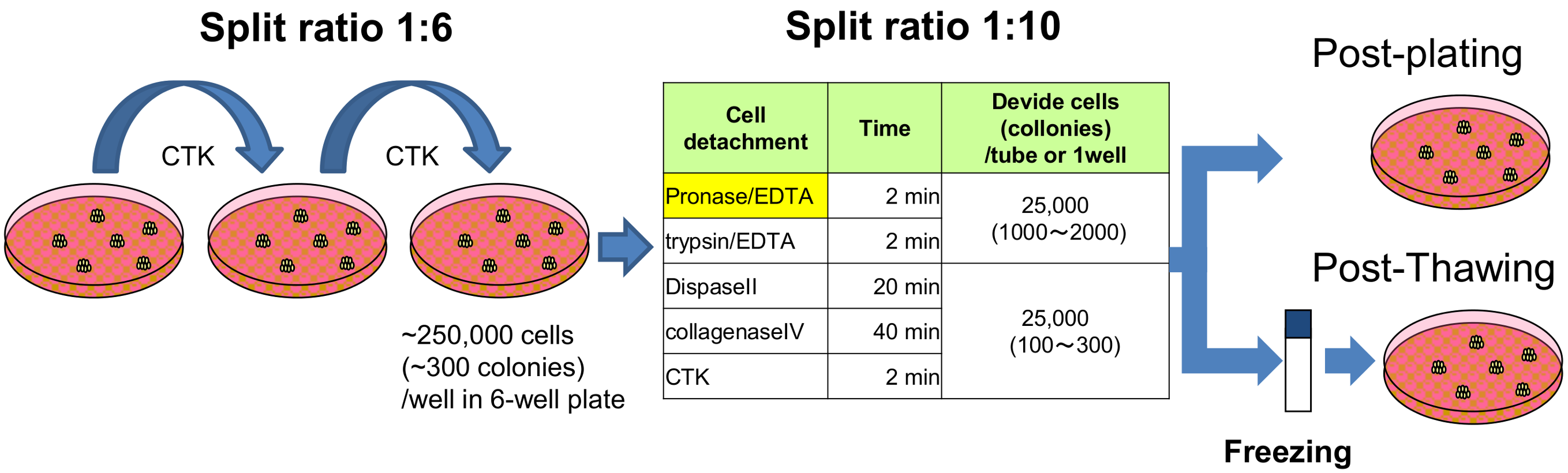

Supplement: Figure S1 — Overview of the protocol for cell passage and the selection of the optimal dissociation buffer for slow-freezing. Schema shows the protocol for regular hPSCs passage with CTK (1∶6 split ratio, left) and selection of the optimal dissociation buffer for slow-freezing (1∶10 split with various dissociation buffers, right). The 1∶10 split ratio was used for this comparison study to score the number of colonies from small cell clumps after trypsin/EDTA or Pronase/EDTA dissociation. (TIF) [file pone.0088696.s001.tif]
